# Evaluation of the Use of Different Sensitive Cardiac Biomarkers in Determining Myocardial Damage in Cows With Subclinical and Clinical Ketoses

**DOI:** 10.1002/vms3.70390

**Published:** 2025-05-05

**Authors:** Murat Uztimür, Cennet Nur Ünal, Meltem Sağiroğlu

**Affiliations:** ^1^ Department of Internal Medicine Faculty of Veterinary Medicine Bingöl University Bingol Turkey; ^2^ Deparment of Physiology Faculty of Veterinary Medicine Fırat University Elazığ Turkey

**Keywords:** BHBA, cattle, high‐sensitivity cardiac troponin I (hs‐cTnI), ketosis, myocardial damage, troponin

## Abstract

**Background:**

It is unclear whether the increase in BHBA concentration damages the myocardium in ketotic cows. Measurement of troponin is the gold standard in myocardial injury.

**Objective:**

The objective of this study was to explore the impact of elevated β‐hydroxybutyrate (BHBA) concentrations on myocardial damage in cows diagnosed with subclinical ketosis (SCK) and clinical ketosis (CK). Additionally, the study aimed to elucidate the relationship between heart‐type fatty acid–binding protein 3 (H‐FABP 3) and high‐sensitivity cardiac troponin I (hs‐cTnI) concentrations, as well as their correlation with various biochemical parameters that assess myocardial and metabolic health.

**Methods:**

The animal material used in this study consisted of 30 postpartum Holstein cows, aged between 2 and 5 years. The study group comprised 10 cows with SCK, 10 with CK and 10 cows in the control group (CG).

**Results:**

The concentration of hs‐cTnI in cows with CK was significantly higher compared to both the CG (*p* < 0.005) and the SCK group (*p* < 0.019). Similarly, the H‐FABP 3 concentration in the CK and SCK groups was significantly elevated compared to the CG (*p* < 0.001). The creatinine kinase myocardial band (CK‐MB) levels were significantly higher in cows with CK compared to the CG (*p* < 0.010). Furthermore, the aspartate aminotransferase (AST) levels in the CK group were significantly elevated compared to the CG (*p* < 0.011). A significant positive correlation was observed between BHBA concentration and the levels of both hs‐cTnI and H‐FABP 3 in ketotic cows. Additionally, significant correlations were found between various biochemical parameters (alanine aminotransferase [ALT], AST, CK‐MB and phosphorus) and the concentrations of H‐FABP 3 and hs‐cTnI.

**Conclusions:**

This study showed that high BHBA concentrations in ketosis cows may indirectly cause myocardial damage. However, future studies need to investigate the relationship between oxidative stress and changes in antioxidant concentrations and troponin.

## Graphical abstract

The main hypothesis of this study is that increased BHBA concentrations in cows with ketosis may lead to myocardial damage. The animal material used in this study consisted of 30 postpartum Holstein cows, aged between 2 and 5 years. The hsTn‐I concentration in cows with CK was statistically significantly higher than both CG (*p* < 0.005) and SCK (*p* < 0.019). H‐FABP 3 concentration of CK and SCK groups was significantly higher than CG (*p* < 0.001). CK‐MB level was significantly higher in cows in CK group than in CG (*p* < 0.010). AST level in the CK group was significantly higher than in CG (*p* < 0.011). This study showed that high BHBA concentration caused myocardial damage in cows with ketosis.

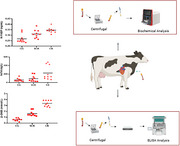

## 1 Introduction

Ketosis is one of the most significant metabolic disorders affecting high‐yielding dairy cows during the pre‐ or postpartum period, primarily caused by negative energy balance (NEB) when energy demands exceed the animal's ability to meet them (Vatnikov et al. 2022). NEB triggers ketogenesis, a process in which free fatty acids oxidized in the liver are converted into ketone bodies, including acetone, acetoacetic acid and β‐hydroxybutyric acid (BHBA) (Gulinski 2021). Early clinical symptoms of ketosis include reduced appetite, lower milk yield, weight loss, pica, reluctance to move and altered faecal consistency, whereas advanced stages are marked by neurological symptoms, such as seizures, aggressive behaviour and excessive salivation (Gordon et al. 2013). This condition leads to significant economic losses for livestock farms due to increased veterinary costs, decreased milk production and impaired reproductive performance (Vatnikov et al. 2022; Gulinski 2021). Moreover, studies have highlighted its adverse effects on vital organs, including the abomasum, uterus, udder and liver, with the severity closely linked to BHBA levels (Duffield et al. 2009; Seifi et al. 2011; Martinez et al. 2012; Xu and Wang 2008). Studies have identified the heart as one of the organs affected by elevated blood BHBA concentrations (Leite Soares et al. 2019; Tharwat et al. 2012). Increased BHBA levels in sheep (Souza et al. 2019) and goats (Tharwat et al. 2012) with pregnancy toxaemia have been shown to cause focal degenerative changes in myocardial contractility and conductivity. A potential explanation for this involves the heightened formation of free radicals, a reduction in antioxidant defences and increased lipolysis, which collectively result in damage to heart cells (Sahoo et al. 2009). Additionally, the rise in non‐esterified fatty acid (NEFA) concentration during the ketoacidosis process, alongside lipolysis, may induce micelle formation within plasma membranes, ultimately causing membrane destabilization and rupture (Moller et al. 2005). Although a recent study (Leite Soares et al. 2019) reported cardiac damage in cows with clinical ketosis (CK), it faced notable limitations in conclusively demonstrating myocardial injury. Heart‐type fatty acid–binding protein 3 (H‐FABP 3) is a cytosolic protein essential for lipid metabolism, including the transport and oxidation of fatty acids, as well as glucose homoeostasis in muscle tissues (Nowowiejska et al. 2022). Furthermore, H‐FABP 3 has been recognized as a promising biomarker for diagnosing myocardial damage in cardiac conditions such as acute myocardial infarction and heart failure. This is due to its release into circulation from damaged cardiomyocytes, providing a reliable indicator of cardiac injury (Baran et al. 2017; Atay et al. 2019; Jirak et al. 2022). In human medicine, cardiac parameters, such as cardiac troponin I (cTnI), creatinine kinase myocardial band (CK‐MB) and aspartate aminotransferase (AST), are routinely used to detect myocardial damage (Atay et al. 2019; Apple et al. 2001). Similarly, in veterinary medicine, these parameters are widely employed to evaluate myocardial injury in cattle, sheep and goats (Leite Soares et al. 2019; Souza et al. 2019; Ünal and Uztimür 2024; Abdelaal et al. 2013). cTnI, a critical cardiac biomarker, binds to actin filaments and is typically present in low concentrations in the cytosol. During cardiomyocyte injury, it is released into the extracellular space, providing key insights into cardiac damage (Wells and Sleeper 2008). The high‐sensitivity cardiac troponin I (hs‐cTnI) assay offers an even greater diagnostic advantage by detecting very low concentrations of cTnI, surpassing the standard‐sensitivity cTnI test in precision (Klüser et al. 2019). Combining hs‐cTnI measurements with H‐FABP 3 concentration analysis in animals with ketosis is considered vital for an early detection of myocardial damage, enabling timely intervention to mitigate adverse effects. A literature review revealed that hs‐cTnI and H‐FABP 3 concentrations were not evaluated in cows with subclinical and CK. The main hypothesis of this study is that increased BHBA concentrations in cows with ketosis may lead to myocardial damage. In line with this hypothesis, we investigated the role of increased BHBA concentration in myocardial damage in cows with subclinical CK and measured hs‐cTnI and H‐FABP 3 concentrations.

## 2 Materials and Methods

This study was commenced following the approval of Bingöl University Animal Experiments Local Ethics Committee (B.U AELEC Date: 2023/01 Decision No: 01/08).

### 2.1 Animals

The study involved 30 postpartum Holstein cows aged between 2 and 5 years, all within the first 21 days postpartum, housed in a semi‐open barn with access to a free‐roaming area. The group consisted of 10 cows with subclinical ketosis (SCK), 10 with CK and 10 healthy cows as a control group (CG). To maintain homogeneity, cows with protozoan infections, such as *Theileria*, *Babesia* or *Anaplasma*, metabolic disorders, including mastitis, laminitis, hypocalcaemia, rumen acidosis or abomasal ulcers, as well as those with a history of abortion, medication use or non‐Holstein breeds, were excluded. All lactating cows were fed a diet comprising soya bean meal, corn silage, bypass oil, alfalfa, sodium and calcium carbonate, whole cottonseed and ad libitum access to vitamins and minerals, prepared as total mixed feeds. The feed's nutritional composition included 61% dry matter, 7.5% crude fat and 5% crude ash.

### 2.2 Body Condition Score (BCS) and Clinical Examination

Before proceeding with further evaluations, all groups of cows involved in the study underwent a comprehensive physical examination, including assessments of respiratory rate (breaths per minute), body temperature (in degrees Celsius) and heart rate (beats per minute). To determine the BCSs of the animals, a visual technique based on a scale ranging from 0 to 5, developed by Edmonson et al. (1989), was utilized.

### 2.3 BHBA Analysis and Study Groups

Postpartum cows were subjected to BHBA analysis every 7 days. Following the morning feeding and milking, blood samples were collected from the *vena jugularis* to determine the BHBA concentration. The BHBA levels were analysed using a handheld electronic device (FreeStyle Optimum Neo, Abbott Diabetes Care Ltd., Witney, UK) along with β‐ketone test strips (FreeStyle Optimum β‐Ketone, Abbott Diabetes Care Ltd.). On the basis of their BHBA concentration, the cows were categorized into three subclasses: CK (blood BHBA: ≥2.6 mmol/L, *n* = 10), SCK (blood BHBA: ≥1.2 mmol/L, *n* = 10) and CG (blood BHBA: <1.2 mmol/L, *n* = 10) (Marutsova et al. 2015; Yáñez et al. 2022).

### 2.4 Blood Sampling and Biochemical Analysis

To determine alanine aminotransferase (ALT), gamma glutamyl transferase (GGT), phosphorus, urea, creatine, triglyceride, high‐density lipoprotein (HDL), low‐density lipoprotein (LDL), very low‐density lipoprotein (VLDL), cholesterol, glucose, H‐FABP 3 and hs‐cTnI levels, we collected 8 mL blood samples from the vena jugularis, using anticoagulant‐free tubes (BD Vacutainer, Plymouth, UK) and following the appropriate technique. After allowing the blood samples to clot for approximately 1 h at room temperature, we separated the serum by centrifugation (Hermle Z 36 HK, Germany) at 3000 rpm for 5 min. The resulting serum samples were stored at −20°C until further analysis. ALT, GGT, phosphorus, urea, creatine, triglyceride, HDL, LDL, VLDL, cholesterol and glucose analyses were carried out using an automated chemistry analyser (Advia 1800, Siemens Healthcare Diagnostics, Malvern, PA, USA). The analysis of serum hs‐cTnI (Siemens Advia Centaur XPT) was performed within 2 months. The within‐run precision coefficient of variation (CV) of the immunoassay was determined by analysing low (mean = 29.24 ng/L) and high (mean = 446.28 ng/L) hs‐cTnI concentrations five times in a row on the same day. CV% for low and high hs‐cTnI concentrations were 6.74% and 9.60%, respectively. For serum H‐FABP 3 analysis, a commercial bovine‐specific ELISA (enzyme‐linked immunosorbent assay) kit (Cattle Heart Fatty Acid Binding Protein ELISA kit, Shanghai Coon Koon Biotech Co. Ltd, China) was utilized. The ELISA was conducted following the test procedure steps specified by the company. In this study, a double‐antibody sandwich ELISA was used, and the optical density was measured at a wavelength of 450 nm using a microplate reader (BioTek Instruments, USA). To ensure the reliability of the study, standards were run in duplicate. The intra‐assay CV for H‐FABP 3 was set at intra‐assay (CV) <7% and inter‐assay (CV) <10%.

### 2.5 Statistical Analysis

The study utilized a sample size of 30 cows, distributed evenly into 3 groups with 10 cows in each group. The determination of this sample size was facilitated by the G*Power software programme (Version 3.1.9.7), with specific parameters set to an alpha error of 0.05, power of 90% and effect size of 0.7. Statistical analysis of the collected data was conducted employing SPSS 26 (IBM SPSS Statistics for Windows, Version 22.0, Armonk, NY: IBM Corp.) and GraphPad Prism (Prism 9 for Windows, version 9). The data were presented as mean ± standard deviation. To assess the normality of the data distribution, the Shapiro–Wilk test was employed. For comparisons between the ketosis group (SCK and CK) and the CG, the Kruskal–Wallis test followed by the Mann–Whitney *U*‐test or the one‐way analysis of variance (ANOVA) followed by the post hoc Tukey multiple comparisons test were applied, as appropriate. In this study, the values of the correlation coefficients of Schober et al. (2018) were interpreted as follows: *r* = 0.00–0.10, negligible correlation; *r* = 0.10–0.39, weak correlation; *r* = 0.40–0.69, moderate correlation; *r* = 0.70–0.89, strong correlation; *r* = 0.90–1.00, very strong correlation. We set the statistical significance level at a *p* value of <0.05 to determine significant differences between the groups.

## 3 Results

**FIGURE 1:**
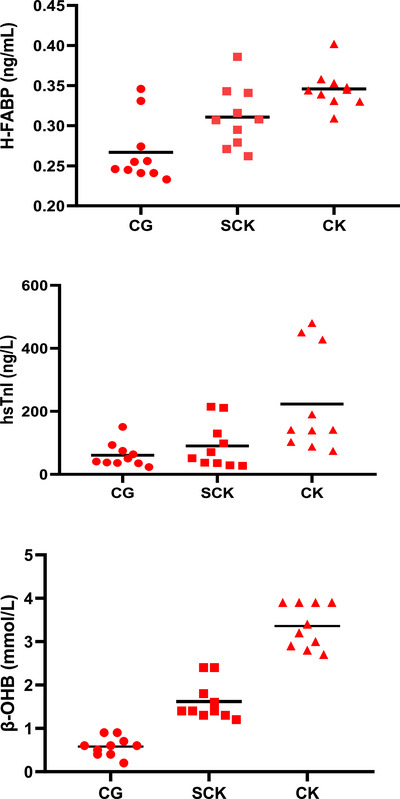
The scatter plot shows the concentration of H‐FABP, hsTnI and BHBA in SCK, CK and CG cows. The black horizontal bar shows the mean value. CG, control group; CK, clinical ketosis; H‐FABP, heart‐type fatty acid–binding protein; hsTnI, high‐sensitivity troponin I; SCK, subclinical ketosis.

The cardiac parameters of cows with SCK, CK and CG are shown in Table 1, and their graphs are shown in Figure 1. H‐FABP 3 concentration of CK and SCK groups was significantly higher than CG (*p* < 0.001). There was also a statistically significant difference between CK and SCK groups (*p* < 0.015). The hs‐cTnI concentration in cows in the CK group was significantly higher than in CG (*p* < 0.005) and SCK (*p* < 0.019). CK‐MB level was significantly higher in cows in CK group than in CG (*p* < 0.010). There was also a statistically significant difference between CK and SCK groups (*p* < 0.001). AST level in the CK group was significantly higher than in CG (*p* < 0.011). There was also a statistically significant difference between CK and SCK groups (*p* < 0.023). No significant difference was determined between ketosis groups and CG in terms of heart frequency (*p* > 0.91), respiratory frequency (*p* > 0.231) and body temperature. In contrast, the BCS of CK cows was significantly higher than that of SCK and CG (0.002). Additionally, the BCS score of SCK was found to be significantly higher than that of CG (*p* < 0.042).

**TABLE 1:** Mean ± standard deviation values of cardiac parameters of subclinical and clinical ketosis and control group cows and statistical differences between groups.

Parameters	SCK	CK	CG	*p*
H‐FABP 3 (ng/mL)	0.31 ± 0.03^a^	0.35 ± 0.02^b^	0.27 ± 0.04^c^	<0.001
hsTnI (ng/L)	90.43 ± 72.45^b^	223.50 ± 161.94^a^	60.52 ± 38.01^b^	0.005
CK‐MB (U/L)	116.16 ± 35.36^b^	253.17 ± 92.97^a^	152.18 ± 59.40^b^	<0.001
AST (U/L) Heart rate (beats/min) Respiratory rate (respiratory rate/minute) Body temperature (°C) BCS	158.70 ± 77.20^b^ 77.20 ± 12.80 35.60 ± 6.09 38.44 ± 0.30 3.12 ± 0.13^a^	291.50 ± 143.93^a^ 86.10 ± 7.03 35.20 ± 7.43 38.43 ± 0.42 3.37 ± 0.27^b^	130.40 ± 98.85^b^ 77.7 ± 12.80 31.30 ± 6.86 38.44 ± 0.27 2.95 ± 0.19^c^	0.010 0.91 0.231 0.979 0.002

*Note*: Data are presented as mean ± standard deviation. Differences between groups with different letters (a–c) in the same row are significant (*p* < 0.05).

Abbreviations: AST, aspartate aminotransferase; BCS, body condition score; CG, control group; CK, clinical ketosis; CK‐MB, creatinine kinase myocardial band; H‐FABP‐3: heart‐type fatty acid–binding protein 3; hsTn‐I, high‐sensitivity troponin I; SCK, subclinical ketosis.

Biochemical values of cows with SCK, CK and CG were shown in Table 2. It was found that the BHBA level of cows in CK and SCK groups was significantly higher than in CG (*p* < 0.001). There was also a statistically significant difference between CK and SCK groups (*p* < 0.001). ALT level of cows in CK and SCK groups was significantly higher than in CG (*p* < 0.013). Although HDL (*p* > 0.279) and cholesterol (*p* > 0.063) levels of cows in CK and SCK groups were lower than CG, no statistically significant difference was found between the groups. There was no significant difference between CK and SCK groups and CG in terms of glucose (*p* > 0.910), LDL (*p* > 0.475), VLDL (*p* > 0.858) and triglyceride (*p* > 0.858) levels. Although phosphorus (*p* > 0.194), GGT (*p* > 0.896), urea (*p* > 0.139) and creatinine (*p* > 0.169) values of cows in CK and SCK groups were higher than CG, no statistically significant difference was found between the groups.

**TABLE 2:** Mean ± standard deviation values of biochemical values of subclinical and clinical ketosis and control group cows and statistical differences between groups.

Parameters	SCK	CK	CG	*p* value
BHBA (mmol/L)	1.62 ± 0.44^a^	3.36 ± 0.50^b^	0.58 ± 0.22^c^	<0.001
ALT (U/L)	23.9 ± 11.23^a^	24 ± 9.13^a^	15.70 ± 6.96^b^	0.013
Glucose (mg/dL)	55.30 ± 10.74	47.70 ± 24.47	49 ± 24.27	0.910
HDL (mg/dL)	56.50 ± 18.40	63.97 ± 17.78	73.69 ± 33.14	0.279
LDL (mg/dL)	7.81 ± 2.60	11.75 ± 8.58	11.47 ± 5.76	0.475
VLDL (mg/dL)	5.38 ± 1.14	5.10 ± 1.32	5.26 ± 0.78	0.858
Triglyceride (mg/dL)	26.90 ± 5.72	25.50 ± 6.60	26.30 ± 3.89	0.858
Cholesterol (mg/dL)	78.30 ± 32.21	92.40 ± 27.98	111.60 ± 59.29	0.063
Phosphor (mg/dL)	5.08 ± 1.0	5.57 ± 1.0	4.75 ± 0.9	0.194
GGT (U/L)	29.80 ± 18.83	28.60 ± 13.23	26.30 ± 11.71	0.896
Urea (mg/dL)	30.20 ± 8.93	37 ± 9.49	29.40 ± 11.86	0.139
Creatine (mg/dL)	0.84 ± 0.19	0.88 ± 0.16	0.74 ± 0.12	0.169

*Note*: Data are presented as mean ± standard deviation. Differences between groups with different letters (a–c) in the same row are significant (*p* < 0.05).

Abbreviations: ALT, alanine aminotransferase; BHBA, beta‐hydroxybutyric acid; CG, control group; CK, clinical ketosis; GGT, gamma‐glutamyl transferase; HDL, high‐density lipoprotein; LDL, low‐density lipoprotein; SCK, subclinical ketosis; VLDL, very low‐density lipoprotein.

**TABLE 3:** Correlation values of the biochemical parameters in the study between high‐sensitivity troponin I (hsTnI) and heart‐type fatty acid–binding protein (H‐FABP).

Parameters	H‐FABP (pg/mL)	hsTnI (ng/L)
BHBA (mmol/L)	0.614^**^	0.450^*^
CK‐MB (U/L)	0.434^*^	—
AST (U/L)	0.415^*^	0.464^**^
ALT (U/L)	0.483^**^	—
Phosphor (mg/dL)	—	0.403^*^
Urea (mg/dL)	—	0.392^*^

Abbreviations: ALT, alanine aminotransferase; AST, aspartate aminotransferase; CK‐MB, creatinine kinase myocardial band.

* *p* < 0.05.

** *p* < 0.01.

The correlation values of the biochemical parameters in the study between hs‐cTnI and H‐FABP 3 are shown in Table 3. In cows with CK and SCK, H‐FABP 3 value was positively correlated with hs‐cTnI, CK‐MB, AST and ALT concentrations at a moderate level and with BHBA concentration at a high level. In cows with CK and SCK, there was a moderately positive significant relationship between hs‐cTnI concentration and BHBA, AST, phosphorus and urea levels.

The BCS value of the cows in the study was 3.25, the average lactation number was 2.1, the average age was 3.5, the average number of seeds per animal was 2.5 and the average monthly milk yield was 23 L.

## 4 Discussion

The primary objective of this study was to investigate the potential development of cardiac damage in cows diagnosed with SCK and CK. Additionally, the research aimed to evaluate the efficacy of novel cardiac biomarkers, H‐FABP 3 and hs‐cTnI, in detecting myocardial injury by analysing their concentration levels. Previous studies have identified cardiac damage in cows with CK, notably in research by Leite Soares et al. (2019). However, the presence of concurrent metabolic diseases, such as left abomasal displacement, metritis, pneumonitis and renal failure, in some subjects of that study raises questions about whether myocardial damage was specifically caused by CK or by other conditions. To address this limitation, the present study exclusively included cows diagnosed solely with ketosis, eliminating confounding factors. The findings demonstrated the release of H‐FABP 3 and hs‐cTnI proteins from cardiomyocyte cells into the peripheral circulation in cows with SCK and CK, providing clear evidence of myocardial damage. Moreover, a significant positive correlation was identified between the concentrations of H‐FABP 3 and hs‐cTnI and the levels of CK‐MB, BHBA, AST, ALT, phosphorus and urea. This research represents the first study to utilize H‐FABP 3 and hs‐cTnI as biomarkers to conclusively demonstrate myocardial damage in cows affected by SCK and CK. The measurement of BHBA is considered the gold standard for diagnosing ketosis in cows (Gulinski 2021; Duffield et al. 2009). Ketosis is categorized into SCK and CK based on blood BHBA levels. However, there is no standardized threshold universally accepted for differentiating between SCK and CK. Some researchers (Goldhawk et al. 2009; Walsh et al. 2007; Marutsova et al. 2015; Yáñez et al. 2022) use blood BHBA concentrations greater than 1.00 or 1.2 mmol/L as a marker for SCK, whereas others (Duffield et al. 2009; Geishauser et al. 2000) set the threshold at >1.4 mmol/L. Similarly, for diagnosing CK, certain studies (Ha et al. 2023; Mellado et al. 2018) use a blood BHBA level >3 mmol/L, whereas others (Marutsova et al. 2015; Issi et al. 2016) accept ≥2.6 mmol/L. In line with the previous research (Duffield et al. 2009; Geishauser et al. 2000; Marutsova et al. 2015; Yáñez et al. 2022), the present study classified ketosis based on blood BHBA concentration, using a threshold of ≥1.2 mmol/L for SCK and ≥2.6 mmol/L for CK (McArt et al. 2013; Leite Soares et al. 2019; Uztimür et al. 2024). The results indicated significantly higher blood BHBA levels in cows with CK (3.36 ± 0.50 mmol/L) and SCK (1.62 ± 0.44 mmol/L) compared to the CG (0.58 ± 0.22 mmol/L). Specifically, cows with CK showed blood BHBA concentrations approximately six times higher than the CG, whereas those with SCK had concentrations roughly three times higher. These findings align with those in existing literature (Issi et al. 2016; Marutsova et al. 2015; Yáñez et al. 2022). The elevated BHBA levels observed in ketosis are likely due to excessive lipid oxidation, a response to NEB in the body (Souza et al. 2020). ALT is a key marker for potential liver cell damage, as its presence in circulation is elevated during liver injury (Abdelaal et al. 2013). ALT is noted for its high sensitivity in diagnosing acute liver damage (Li et al. 2020; Abdelaal et al. 2013). Studies by Du et al. (2017) and Li et al. (2016) have shown that ALT levels are higher in cows with ketosis when compared to CGs. In the present study, ALT levels were significantly increased in cows with SCK and CK relative to the CG, suggesting liver damage. The elevated ALT concentrations observed in these cows are likely due to increased lipolysis associated with NEB, which leads to fat accumulation in the liver and subsequent cell degeneration (Abdelaal et al. 2013). AST and CK‐MB levels are markedly elevated in cases of skeletal–muscular system damage (Souza et al. 2020; Issi et al. 2016; Uztimür et al. 2024). Additionally, AST activity is used in conjunction with other enzymes to indicate liver damage. Studies on cows with ketosis (Leite Soares et al. 2019) and goats with pregnancy toxaemia (Souza et al. 2019, 2020; Uztimür and Ünal 2024) have reported significantly increased AST and CK‐MB activities. In the present study, AST levels were significantly elevated in cows with CK (291.50 ± 143.93 U/L) and SCK (158.70 ± 77.20 U/L) compared to the CG (130.40 ± 98.85 U/L). Additionally, CK‐MB levels were significantly higher in cows with both CK (253.17 ± 92.97 U/L) and SCK (116.16 ± 35.36 U/L) compared to the CG (152.18 ± 59.40 U/L). The increase in AST and CK‐MB activity is thought to be linked to skeletal muscle, heart and liver deterioration (Souza ve ark. 2020; Kırbaş et al. 2021).

Studies have indicated a 96.4% antigenic similarity in cTnI between humans and cattle (O'Brien et al. 1997). Due to this significant antigenic similarity, human‐derived hs‐cTnI concentrations were utilized for measurements in this research. Additionally, various devices originally designed for human‐specific cTnI measurements have been adapted for use in other animals, such as cats, dogs, goats, cattle and horses (Kırbaş et al. 2021; Hanås et al. 2022; Varga et al. 2009; Nath et al. 2012; Ünal and Uztimür 2024; Winter et al. 2017; Souza et al. 2020). The International Federation of Clinical Chemistry and Laboratory Medicine recommends that acceptable test precision should have a CV of <10% (Panteghini et al. 2001; Panteghini et al. 2004). For instance, Tumer and Şafak (2022) determined that the mean serum cTnI concentrations of 0.014 and 2.302 ng/mL in sheep serum samples tested by Advia Centaur TnI‐Ultra had intra‐assay CVs of 8.1% and 4.14%, respectively. In bovine serum, Varga et al. (2009) reported a mean cTnI concentration range of 0.2–30 ng/mL, with intra‐assay precision values of 3.09% and 4.84%. In line with these prior findings, the current study showed that mean hs‐cTnI concentrations in serum samples from Advia Centaur XPT were 29.24 and 446.28 ng/L, with intra‐assay precisions of 6.74% and 9.60%, respectively, confirming they fall within the acceptable limit of <10% CV.A. There is only one study in which cTnI concentration was specifically determined in cows with ketosis (Leite Soares et al. 2019). This study found a significant increase in cTnI concentration in cows with CK, suggesting cardiac damage. Souza et al. (2019) conducted research on sheep with pregnancy toxaemia and reported a correlation where an increase in BHBA concentration was associated with higher cTnI levels, indicating myocardial damage. They further noted that cardiac damage contributed to higher mortality rates in sheep that did not survive pregnancy toxaemia. In a subsequent study by the same researchers (Souza et al. 2020), they observed increased cTnI levels in goats with pregnancy toxaemia, again indicating cardiac damage in this metabolic condition. Supporting these findings, Tontis and Zwahlen (1987) identified focal degenerative changes in myocardial cells’ contraction and conduction in sheep with pregnancy toxaemia, confirming cardiac damage. In the current study, hs‐cTnI concentration was significantly elevated in cows with SCK (90.43 ± 72.45 ng/L) and CK (223.50 ± 161.94 ng/L) compared to the CG (60.52 ± 38.01 ng/L). Additionally, moderately significant relationships were found between hs‐cTnI concentrations and BHBA (*r* = 0.450, *p* = 0.013), AST (*r* = 0.464, *p* = 0.010), phosphorus (*r* = 0.403, *p* = 0.027), urea (*r* = 0.392, *p* = 0.032) and H‐FABP 3 (*r* = 0.368, *p* = 0.045). In pregnancy toxaemia, an increase in blood BHBA concentration leads to oxidative stress due to the severity of lipolysis, resulting in a decrease in antioxidant activity. Free radicals exhibit cytotoxic effects through the peroxidation of membrane phospholipids, leading to structural changes, increased membrane permeability and cell death in cardiac cells. Therefore, it is thought that the concentration of BHBA indirectly affects cardiac damage in pregnancy toxaemia (Souza et al. 2019, 2020; Leite Soares et al. 2019). These findings align with existing literature, confirming a significant correlation between BHBA and hs‐cTnI (*r* = 0.450, *p* = 0.013) (Leite Soares et al. 2019; Souza et al. 2020). H‐FABP, a cytoplasmic protein residing within cardiac cells, plays a pivotal role in the uptake, transportation and oxidation of fatty acids (Kleine et al. 1992). In a study involving individuals with acute myocardial infarction, it was ascertained that the concentration of H‐FABP was markedly elevated (Baran et al. 2017). Another investigation revealed that H‐FABP levels exhibited a noteworthy diagnostic characteristic in humans with acute coronary syndrome, surpassing those of the healthy group (Gururajan et al. 2010). Only a solitary study (Yıldız et al. 2019) has addressed the determination of H‐FABP levels in ruminants. Within this inquiry conducted by Yıldız et al. (2019) concerning cattle afflicted with traumatic pericarditis, it was reported that the concentration of H‐FABP showed a significant increase when compared to the healthy group. Moreover, the use of this biomarker proved beneficial in assessing cardiac damage. As demonstrated by the present study, the concentration of H‐FABP 3 exhibited a substantial increase in cows afflicted with both CK (0.35 ± 0.02 ng/mL) and SCK (0.31 ± 0.03 ng/mL), as opposed to CG (0.27 ± 0.04 ng/mL). Moderate correlations were observed between the concentration of H‐FABP 3 and the levels of hs‐cTnI (*r* = 0.368, *p* = 0.045), CK‐MB (*r* = 0.434, *p* = 0.016), AST (*r* = 0.415, *p* = 0.023) and ALT (*r* = 0.483, *p* = 0.007). Furthermore, modest correlation was noted between H‐FABP and BHBA (*r* = 0.614, *p* = 0.001) levels. It has been reported that the cytotoxic impact of free radicals, through peroxidation of membrane phospholipids, induces cell death, heightens membrane permeability and causes disruption of the plasma membrane of myocardial cells, leading to the release of cardiac cells into circulation and formation of micelles (Gerede et al. 2016; El‐Deeb 2015; Lima and Abdalla 2001).

There are many different factors that affect the formation of cardiac damage (Mellado et al. 2018; Schultze et al. 2008). However, the presence of cardiac damage has been demonstrated in many studies by measuring only troponin concentration (Leite Soares et al. 2019; Souza et al. 2019, 2020). In the studies conducted by Leite Soares et al. (2019), Souza et al. (2019) and Souza et al. (2020), oxidative stress or oxidant load was not analysed, and they attributed myocardial damage to the increase in blood BHBA concentration. In our study, we do not claim that the increase in troponin is directly caused by the concentration of BHBA or that BHBA is the sole culprit for cardiac damage, and we have included an explanation in the article stating that it has an indirect effect.

This study has few limiting factors. Although clinical auscultatory examinations were conducted on groups of cows with SCK and CK, no cardiac murmurs, churning or friction findings were observed. However, echocardiographic examinations were regrettably not feasible for the groups of cows with SCK, CK and those in the CG. Although the sample size of the study was determined as 30 cows for all groups (10 cows in each group) with the help of the G*Power (Version 3.1.9.7) software programme with an alpha error of 0.05, a power of 90% and an effect size of 0.7, the study should be conducted with a larger number of animals. The acute phase response and inflammation were not determined in this study. However, it is known that both inflammation and acute phase response occur in cows in ketosis.

In conclusion, this study demonstrated the presence of myocardial damage in ketotic Holstein cows. It was found that there is a significant positive correlation between cardiac biomarkers (hs‐cTnI and H‐FABP 3) and blood levels of BHBA, CK‐MB, AST, ALT, phosphorus and urea. These biomarkers show promise for prognostic applications in assessing heart disease in ketotic cows. However, to fully understand the mechanisms behind the release of hs‐cTnI and H‐FABP 3 from the myocardium into the peripheral circulation, as well as to evaluate the reversibility of myocardial damage after ketosis treatment, additional research involving larger populations and more extensive numbers of animals is necessary.

## Author Contributions

**Murat Uztimür**: design, writing and literature search of the study. **Murat Uztimür** and **Cennet Nur Ünal**: sample collection and sending for analysis. **Meltem Sağiroğlu**: statistical analysis of the data and evaluation of the article.

## Ethics Statement

The research received approval from the Bingöl University Experimental Animals Ethics Committee (Date and Number: 19.01.2023‐01/08).

## Conflicts of Interest

The authors declare no conflicts of interest.

### Peer Review

The peer review history for this article is available at https://www.webofscience.com/api/gateway/wos/peer‐review/10.1002/vms3.70390.

## Data Availability

The data that support the findings of this study are available from the corresponding author upon reasonable request.
